# Supplementary material for: Environmental versus phylogenetic controls on leaf nitrogen and phosphorous concentrations in vascular plants
Source: Nat Commun. 2024 Jun 24;15:5346. doi: 10.1038/s41467-024-49665-4 (PMC11196693; doi:10.1038/s41467-024-49665-4)
Supplement: Supplementary file 1 — Supplementary Information [file 41467_2024_49665_MOESM1_ESM.pdf]

Supplementary for "Environmental versus phylogenetic controls on leaf nitrogen and phosphorous concentrations of vascular plants"

Di Tian[1,2,3*], Zhengbing Yan[4], Bernhard Schmid[5,6], Jens Kattge[7,8], Jingyun Fang[6], Benjamin D. Stocker[2,3,9,10*]

[1] State Key Laboratory of Efficient Production of Forest Resources, Beijing Forestry University, Beijing 100083, China

[2] Institute of Agricultural Sciences, Department of Environmental Systems Science, ETH, Universitätsstrasse 2, Zürich 8092, Switzerland

[3] Swiss Federal Institute for Forest, Snow and Landscape Research WSL, Zürcherstrasse 111, 8903 Birmensdorf, Switzerland

[4] State Key Laboratory of Vegetation and Environmental Change, Institute of Botany, Chinese Academy of Sciences, Beijing 100093, China

[5] Department of Geography, Remote Sensing Laboratories, University of Zürich, Zürich 8006, Switzerland

[6] Institute of Ecology, College of Urban and Environmental Sciences, Peking University, Beijing 100871, China

[7] Max-Planck-Institute for Biogeochemistry, Hans-Knöll Street 10, Jena 07745, Germany

[8] iDiv - German Centre for Integrative Biodiversity Research Halle-Jena-Leipzig, Puschstraße 4 04103 Leipzig, Germany

[9] Institute of Geography, University of Bern, Hallerstrasse 12, Bern 3012, Switzerland

[10] Oeschger Centre for Climate Change Research, University of Bern, Falkenplatz 16, Bern 3012, Switzerland

[*]**Corresponding authors:**

Di Tian: tiandi@bjfu.edu.cn

Benjamin D. Stocker: benjamin.stocker@unibe.ch

Supplementary Table 1. Climatic variables and climate-related environmental variables used for our analysis.

Abbreviation	Unit	Meaning	From
MAT	℃	Mean annual temperature	WorldClim[1]
MATgs	℃	Mean temperature during growing season	WorldClim[1]
tmmonthmin	℃	Minimal monthly temperature, i.e., temperature of the coldest month	WorldClim[1]
Tmmax	℃	Maximal monthly temperature, temperature of the warmest month	WorldClim[1]
Ndaysgs	day	Number of days with daily temperature above 0˚C (TMP0nb) or 5˚C (TMP5nb)	WorldClim[1]
MAI	umol m^{-2} s^{-1}	Annual mean daily irradiance (PPFD)	WorldClim[1]
MAIgs	umol m^{-2} s^{-1}	Growing season mean daily irradiance	WorldClim[1]
MAP	mm	Mean annual summed precipitation	WorldClim[1]
Pmmin	mm	Precipitation of the driest month	WorldClim[1]

MAPgs	mm	Mean growing season summed precipitation	WorldClim[1]
Mav	kpa	Mean daytime VPD	WorldClim[1]
Mavgs	kpa	Mean daytime VPD during the growing season	WorldClim[1]
Alpha	-	Annual mean ratio of actual over potential evapotranspiration	Davis et al. 2017[2]
V_{cmax25}	mol C m^{-2} d^{-1}	Maximum rate of carboxylation, normalized to 25 ◦C	Estimated by the P-model[3] (Stocker et al. 2020)
J_{max25}	mol C m^{-2} d^{-1}	Electron transport (Jmax) for ribulose-1,5-bisphosphate (RuBP) regeneration	Estimated with the P-model[3] (Stocker et al. 2020)
Gs	m^{-2} s^{-1}	Multi-day average stomatal conductance	Estimated with the P-model[3] (Stocker et al. 2020)
Aet	mm	Mean annual total evapotranspiration	Estimated using Priestly-Taylor[2] (Davis et al. 2017)
AI	-	Aridity index, precipitation over potential evapotranspiration	Estimated using Priestly-Taylor[2] (Davis et al. 2017)
cwdx80	mm	80-year maximum cumulative water deficit	Stocker et al. 2023[4]

ndep	g m^{-2} yr^{-1}	atmospheric N deposition rate	Established by Lamarque et al. (2011)[5]
CO_2	ppm	atmospheric CO_2 concentration	Extracted from the records of Mauna Loa Observatory[6]
Elv	m	Elevation of the sampling site	Extracted from ETOPO1[7] (https://www.ncei.noaa.gov/)
CTI	-	The Compound Topography Index	Marthews et al. (2015)[8]

Supplementary Table 2. Detailed information of the soil variables used in our analysis.

Soil property	Abbreviation	Database	Name in the database	Soil layer	Unit
Soil bulk density	BD	HWSD	T_BULK_DENSITY	0-30 cm	kg dm^{-3}
Available water storage capacity	AWC	HWSD	AWC_CLASS		mm m^{-1}
Topsoil Clay Fraction	Clay	HWSD	T_CLAY		% wt.
Topsoil Silt Fraction	Silt	HWSD	T_SILT		% wt.
Topsoil Sand Fraction	Sand	HWSD	T_SAND		% wt.
Topsoil Gravel Content	Gravel	HWSD	T_GRAVEL		%vol.
Topsoil pH (H2O)	pH	HWSD	T_PH_H2O		-log(H+)
Total exchangeable bases	TEB	HWSD	T_TEB		cmol kg-1

Topsoil Base Saturation	BS	HWSD	T_BS		%
Cation exchange capacity	CEC_{soil}	HWSD	T_CEC_SOIL		cmol kg^{-1}
Cation exchange capacity of clay size fraction	CEC_{clay}	HWSD	T_CEC_CLAY		cmol kg^{-1}
Salinity measured by the electrical conductivity of the soil	ECE	HWSD	T_ECE		dS m^{-1}
Topsoil Sodicity (ESP= Na*100/CECsoil)	ESP	HWSD	T_ESP		%
Topsoil Calcium Carbonate content	CACO3	HWSD	T_CACO3		% weight
Topsoil Organic Carbon Content (the percentage of organic carbon in topsoil)	SOC	HWSD	T_OC		% weight

Exchangeable aluminium percentage (as % of ECEC)	ALSA	WISE30sec	Aluminium saturation	0-40 cm	%
Soil total nitrogen content	TOTN	WISE30sec	Total nitrogen		g kg^{-1}
Organic carbon content	ORGC	WISE30sec	Organic carbon content		g kg^{-1}
Soil C:N ratios	C:N	WISE30sec	C:N ratio		g g^{-1}
Soil phosphorus using the Bray1 method	PBR	GSDE	P_Bray_method	0-28.9 cm	ppm of weight
Total phosphorus content	TP	GSDE	Total_P		% of weight
Total potassium content	TK	GSDE	Total_K		% of weight

Supplementary Table 3. ANOVA table showing the results of variance partition via linear models (LMs) and linear mixed model (LMM) for leaf N. Sum of squares for each predictor in the three different models are shown in the table. Model1: LM with species identity first and the selected environmental variables (i.e., ALSA, mav, elv, co2, tmonthmin, mai and ndep) last. Model2: LM with the selected environmental variables first and species identity last. Model3: LMM with the selected environmental variables as fixed-effect terms and species identity as random-effects term.

**Predictor**	**Model1 (Species first)**	**Model2 (Species last)**	**Model3 (Species random)**
Species	164.07	132.89	NA
ALSA	0.72	0.13	0.52
mav	1.24	1.48	0.64
Elv	0.01	0.81	0.07
co2	1.73	17.80	2.12
tmonthmin	0.08	14.52	2.12
mai	1.56	0.34	1.62
ndep	0.02	1.53	0.01
Residuals	68.67	68.67	NA

Supplementary Fig. 1 Variation partitioning of species identity versus site on leaf N and P concentrations and N:P ratio, *measured by the linear model with the two fitting sequences species ID + Site ID and Site ID + species ID. Site ID comprises all selected environmental variables and additional variation between sites not explained by the selected environmental variables. See also Supplementary Table 3 above.*

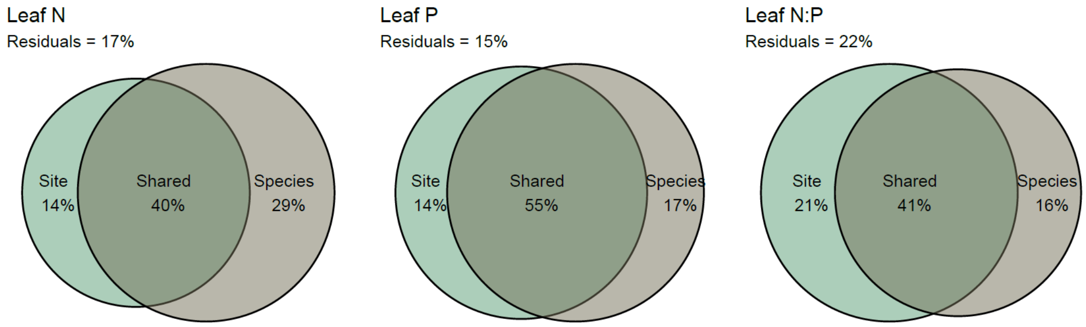

Supplementary Fig. 2 Distribution of sites *from which leaf N and P data were sourced, plotted in climatic space defined by mean annual temperature (x-axis) and mean annual precipitation (y-axis). Domains in the climatic space, aligning with major biomes, are indicated by different colors based on the R package plotbiomes (Ştefan & Levin 2018)*[9].

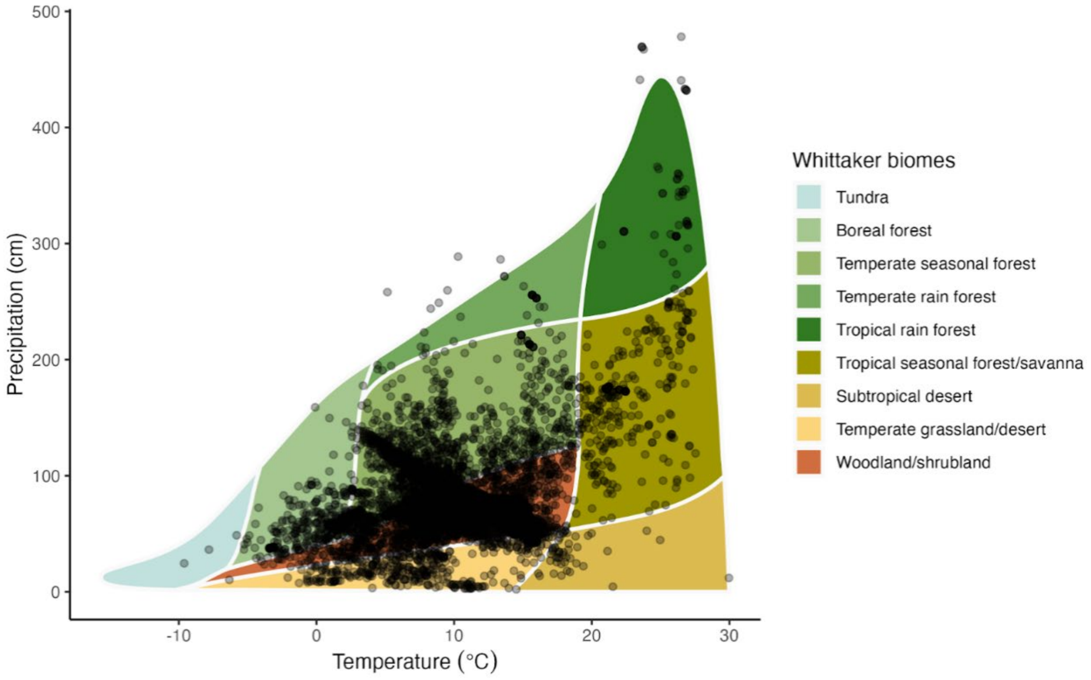

Supplementary Fig. 3 ***Correlation between species-specific ranges in leaf N and P****. r is the Pearson correlation coefficient. Here, each dot represents one species and species are selected as used in the trait gradient analysis, requiring that species occurred in at least five sites where at least five different species were recorded. Additionally, data were subset to exclude species for which the slope in the trait gradient analysis was below -5 or above 5. This retained 324 species.*

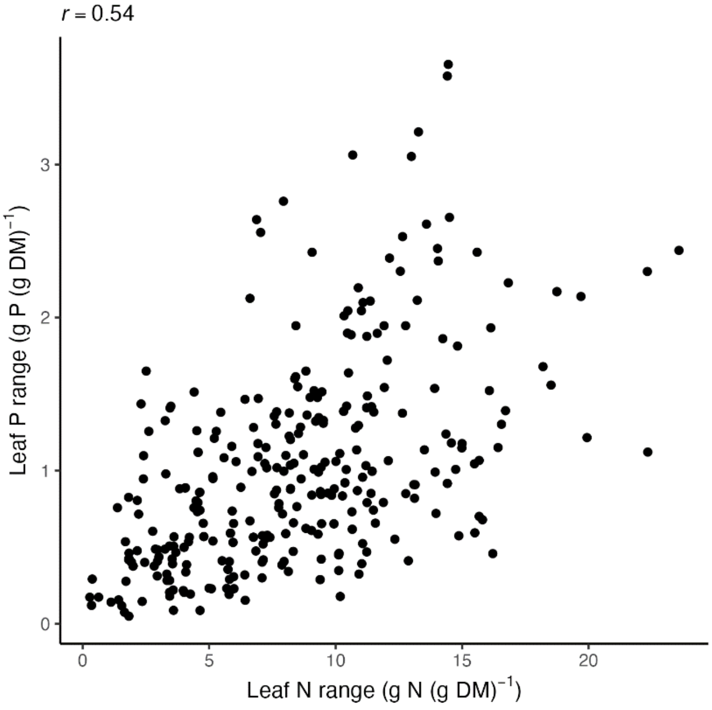

Supplementary References

1. Fick, S. E. & Hijmans, R. J. WorldClim 2: new 1-km spatial resolution climate surfaces for global land areas. *Int. J. Climatol.* **12**, 4302-4315 (2017).
2. Davis, T. W. et al. Simple process-led algorithms for simulating habitats (SPLASH v. 1.0): robust indices of radiation, evapotranspiration and plant-available moisture. *Geosci. Model Dev.* **10**, 689-708 (2017).
3. Stocker, B. D. et al. P-model v1. 0: an optimality-based light use efficiency model for simulating ecosystem gross primary production. *Geosci. Model Dev.* **13**, 1545-1581 (2020).
4. Stocker, B. D. et al. Global patterns of water storage in the rooting zones of vegetation. *Nat. Geosci.* **16**, 250-256 (2023).
5. Lamarque J. F. et al. Global and regional evolution of short-lived radiatively-active gases and aerosols in the Representative Concentration Pathways. *Clim. Change* **109**, 191-212 (2011).
6. Keeling, R. F., Walker, S. J., Piper, S. C. & Bollenbacher, A. F. Exchanges of atmospheric CO_2 and $^{13}CO_2$ with the terrestrial biosphere and oceans from 1978 to 2000. Global aspects, SIO Reference Series, No. 01-06, Scripps Institution of Oceanography, San Diego: 88 (2001).
7. Amante, C. & Eakins, B. W. ETOPO1 1 arc-Minute global relief model: procedures, data sources and analysis. *NOAA Technical Memorandum NESDIS NGDC* **24,** (2009).
8. Marthews, T., Dadson, S., Lehner, B., Abele, S. & Gedney, N. High-resolution global topographic index values for use in large-scale hydrological modelling. *Hydrol. Earth Syst. Sci.* **19**, 91-104 (2015).
9. Ştefan V, Levin S. Plotbiomes: R package for plotting Whittaker biomes with ggplot2 (v1.0.0). *Zenodo*. https://doi.org/10.5281/zenodo.7145245. (2018).
